# PhosBoost: Improved phosphorylation prediction recall using gradient boosting and protein language models

**DOI:** 10.1002/pld3.554

**Published:** 2023-12-20

**Authors:** Elly Poretsky, Carson M. Andorf, Taner Z. Sen

**Affiliations:** ^1^ Agricultural Research Service, Crop Improvement and Genetics Research Unit U.S. Department of Agriculture Albany CA United States; ^2^ Agricultural Research Service, Corn Insects and Crop Genetics Research U.S. Department of Agriculture Ames IA United States; ^3^ Department of Computer Science Iowa State University Ames IA United States; ^4^ Department of Bioengineering University of California Berkeley CA United States

## Abstract

Protein phosphorylation is a dynamic and reversible post‐translational modification that regulates a variety of essential biological processes. The regulatory role of phosphorylation in cellular signaling pathways, protein–protein interactions, and enzymatic activities has motivated extensive research efforts to understand its functional implications. Experimental protein phosphorylation data in plants remains limited to a few species, necessitating a scalable and accurate prediction method. Here, we present PhosBoost, a machine‐learning approach that leverages protein language models and gradient‐boosting trees to predict protein phosphorylation from experimentally derived data. Trained on data obtained from a comprehensive plant phosphorylation database, qPTMplants, we compared the performance of PhosBoost to existing protein phosphorylation prediction methods, PhosphoLingo and DeepPhos. For serine and threonine prediction, PhosBoost achieved higher recall than PhosphoLingo and DeepPhos (.78, .56, and .14, respectively) while maintaining a competitive area under the precision‐recall curve (.54, .56, and .42, respectively). PhosphoLingo and DeepPhos failed to predict any tyrosine phosphorylation sites, while PhosBoost achieved a recall score of .6. Despite the precision‐recall tradeoff, PhosBoost offers improved performance when recall is prioritized while consistently providing more confident probability scores. A sequence‐based pairwise alignment step improved prediction results for all classifiers by effectively increasing the number of inferred positive phosphosites. We provide evidence to show that PhosBoost models are transferable across species and scalable for genome‐wide protein phosphorylation predictions. PhosBoost is freely and publicly available on GitHub.

## 1 INTRODUCTION

Protein phosphorylation is one of the most widespread and important post‐translational modifications that play a pivotal role in the regulation of protein function and cellular pathways (Nishi et al., 2014), and has been extensively studied in plants (Zhang et al., 2023). Through the covalent addition of a phosphate group to specific amino acids, predominantly serine (Ser, S), threonine (Thr, T), or tyrosine (Tyr, Y), protein phosphorylation alters various aspects of protein function, including activity, subcellular localization, stability, and interactions with other proteins or ligands (Álvarez‐Salamero et al., 2017). Its involvement spans a wide array of cellular functions, such as cell signaling, metabolism, development, and resistance to biotic and abiotic stress (Chaudhuri et al., 2015; Dressano et al., 2020; Humphrey et al., 2015; Kim et al., 2009; Nishi et al., 2014; Oh et al., 2009; Ryu et al., 2007; Wang et al., 2013; Zhao & Guo, 2011). The dysregulation of protein phosphorylation has been closely linked to various diseases and is also the target of effectors that can enhance pathogenic virulence (Ardito et al., 2017; Toruño et al., 2016). Often, multiple residues within the same protein will undergo phosphorylation that can have independent, synergistic, or antagonistic functions, revealing the existence of complex “phosphocodes” (Pejaver et al., 2014; Zhang et al., 2023) while crosstalk between different post‐translational modifications can fine‐tune cellular responses (Vu et al., 2018). Cross‐species and ‐protein family comparisons have shown that functional phosphosites are more likely to be conserved, suggesting that phosphorylation information is transferable to the studies of other species (Chaudhuri et al., 2015). Thus, protein phosphorylation has emerged as a powerful target for developing disease treatments and manipulation for enhanced crop yields (Gough & Sadanandom, 2021; Yang et al., 2010).

Protein phosphorylation is among the most studied post‐translational modifications using high‐throughput mass‐spectrometry‐based methods, making a large amount of experimental protein phosphorylation data available from a variety of species, tissues, developmental stages, and conditions (Dou et al., 2021; Xue et al., 2022; Yu et al., 2023), but is relatively understudied in plant species besides *Arabidopsis thaliana* (Meng et al., 2022; Xue et al., 2022). Conservation of phosphosites within and across species has been observed offering a complementary approach for improving phosphosite detection and annotation based on straightforward sequence similarity approaches (Amanchy et al., 2007; Chaudhuri et al., 2015; Maathuis, 2008; Tan et al., 2009). The large increase in the amount of experimental protein phosphorylation data facilitated the development of a variety of machine‐ and deep‐learning‐based protein phosphorylation classifications (Luo et al., 2019; Wang et al., 2017; Zuallaert et al., 2022). For example, classical machine learning algorithms such as random forests (Ismail et al., 2016; Liu et al., 2022; Wei et al., 2017), support vector machines (Dou et al., 2014; Jamal et al., 2021), and gradient boosting trees (Maiti et al., 2020) have been used for protein phosphorylation prediction. More recently, deep learning algorithms such as convolutional neural networks (Guo et al., 2020; Luo et al., 2019; Wang et al., 2017; Zuallaert et al., 2022) and long short‐term memory networks (Lv et al., 2021; Thapa et al., 2021) enabled learning directly from protein sequences, making substantial improvements in protein phosphorylation prediction.

In the absence of kinase specificity and targeted consensus sequences, kinase promiscuity adds to the challenge of protein phosphorylation prediction (Friso & Van Wijk, 2015). Different types of features have been used for protein phosphorylation prediction, often derived from biophysical properties, such as solvent accessibility and disorder score, and structural features, such as secondary structure (Y. Dou et al., 2014; Gao et al., 2010; Jamal et al., 2021). Improvements in deep learning classification methods enabled learning directly from the sequences surrounding the phosphosites without the need for complex feature representations (Luo et al., 2019; D. Wang et al., 2017; Wang et al., 2022). More recently, advances made in natural language processing enabled the development of protein language models (pLMs). By pre‐training on vast numbers of protein sequences, pLMs learned inherent properties encoded within protein sequences, revolutionizing multiple fields of protein research (Bordin et al., 2023; Elnaggar et al., 2021; Ofer et al., 2021; Rives et al., 2021). Most importantly, pLMs capture both short and long‐range functional and biophysical properties of each amino acid within a given protein as an encoded numerical vector, known as vector embeddings, that can be directly used by machine‐ and deep‐learning classifiers (Littmann et al., 2021). The utilization of pLM‐based vector embeddings has proven effective in predicting structural and biochemical properties such as secondary structures, solvent accessibility, and ligand binding residues (Ilzhöfer et al., 2022; Weissenow et al., 2022). The use of pLMs has also been used to develop new methods with improved performance for general and kinase‐specific protein phosphorylation prediction (Zhou et al., 2023; Zuallaert et al., 2022). Recent advances in protein phosphorylation prediction, employing convolutional neural networks with pLM‐based vector embeddings, demonstrated the potential of pLMs in predicting general protein phosphorylation, while also being applicable to other post‐translational modifications (Zuallaert et al., 2022).

Compared to Ser and Thr phosphorylation, the prediction of Tyr phosphorylation poses additional challenges, partially due to a smaller amount of experimental data leading to a relatively higher label imbalance (Doll & Burlingame, 2015; La Fuente et al., 2009; Silva‐Sanchez et al., 2015). Compared to animals, plants completely lack dedicated Tyr‐specific kinases and rely on dual‐specificity Ser/Thr and Tyr kinases for all Tyr phosphorylation, leading to a particularly imbalanced Tyr phosphorylation data in plants (Ghelis, 2011; La Fuente et al., 2009). Thus, while prediction of Tyr phosphorylation poses a challenge in both animals and plants, the challenge is expected to be more acute in plants. Despite the lack of dedicated Tyr kinases in plants, Tyr phosphorylation is known to regulate the function of multiple proteins involved in a variety of essential biological processes (Ghelis, 2011; Mühlenbeck et al., 2021). Consequently, improving the prediction accuracy of Tyr phosphorylation holds great potential for identifying functional phosphosites for a better understanding of cellular signaling and regulatory processes.

Here, we present the newly developed PhosBoost, a machine‐learning classification method that uses pLMs with a stacking classifier composed of CatBoost (Prokhorenkova et al., 2018) gradient‐boosting tree‐based ensemble base classifiers to predict protein phosphorylation directly from protein sequences. We trained and evaluated PhosBoost on a large set of experimentally derived protein phosphorylation data obtained from the plant post‐translational modification database, qPTMplants (Xue et al., 2022). Compared to existing protein phosphorylation prediction methods, PhosBoost consistently achieves higher recall, albeit at reduced or comparable precision for Ser/Thr prediction. PhosBoost provided both higher recall and precision for Tyr prediction. PhosBoost also produces probability scores that are more informative and better reflect the confidence in the protein phosphorylation prediction. To improve phosphosite annotation and reduce phosphosite label uncertainty, we supplemented PhosBoost predictions with a DIAMOND (Buchfink et al., 2021) pairwise alignment analysis step that annotates phosphosites matching experimentally derived data. We provide evidence to show that PhosBoost models are transferable across species and scalable for genome‐wide protein phosphorylation predictions, allowing for the incorporation of PhosBoost prediction results directly in the genome browser to facilitate accessibility. Our results show that PhosBoost is a competitive method for protein phosphorylation predictions, particularly when higher recall and genomic coverage of phosphosites are prioritized.

## 2 RESULTS

### 2.1 Overview of the protein phosphorylation data used to develop PhosBoost

Protein phosphorylation data was obtained from qPTMplants, a comprehensive database for high‐throughput plant post‐translational modification experimental data (Xue et al., 2022). The qPTMplants database includes protein phosphorylation data for over 30 plant species collected from different experiments, representing different organs, developmental stages, and conditions (Xue et al., 2022). Because of the predominance and relative saturation of the *A. thaliana* protein phosphorylation data (Figure S1A‐C), we focused on the *A. thaliana* dataset for model training, hyperparameter tuning, and performance benchmarking. After collecting all experimentally derived positive phosphosites, the remaining Ser, Thr, and Tyr residues were collected to form the negative phosphosite dataset. Random stratification was used on the individual Ser, Thr, and Tyr datasets to generate the training, validation, and test sets using a 60%–20%–20% split, respectively. The Ser and Thr phosphosites were then combined for training and testing a combined binary S/T classification model.

### 2.2 Description of the PhosBoost protein phosphorylation classifier

**FIGURE 1:**
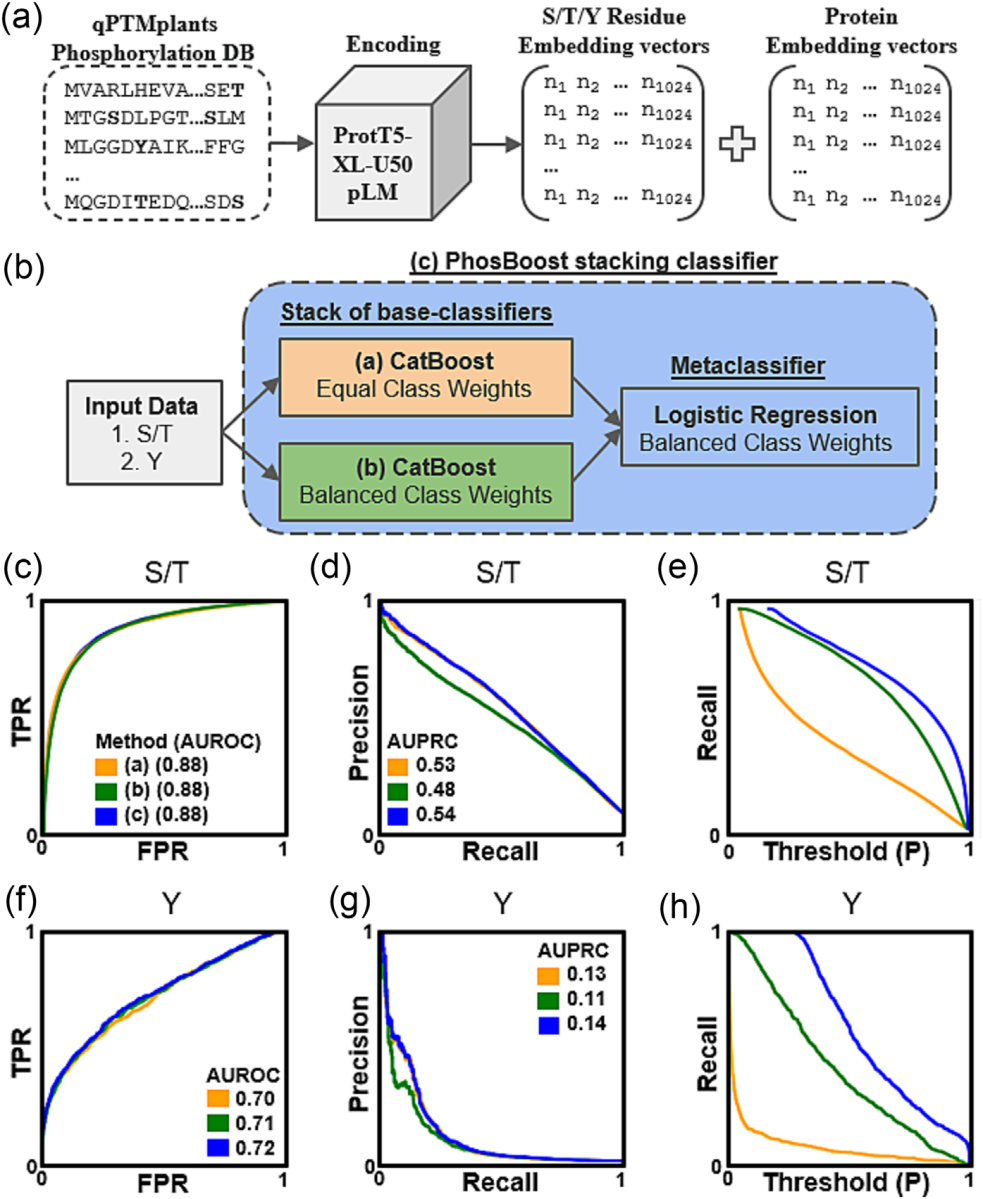
Overview of the PhosBoost protein phosphorylation classification workflow. (a) Protein phosphorylation data from the qPTMplants database were encoded by the pre‐trained ProtT5‐XL‐U50 pLM to generate the embedding vector data for all serine (S), threonine (T), and tyrosine (Y) residues in addition to the protein‐wise average embedding vector. (b) The input data for all *A. thaliana* phosphosites was used to generate two separate binary classifiers: (1) S/T model and (2) Y model, trained using a stacking classifier, termed PhosBoost. The stacking classifier is composed of two CatBoost base classifiers: (a) with equal class weights and (b) balanced class weights. A logistic regression metaclassifier combined the predicted probability scores of (a) and (b) to produce (c) PhosBoost. **(c‐e)** The performances of the independently trained (a, orange) and (b, green) CatBoost classifiers were compared with the performance of the PhosBoost stacking classifier (c, blue), showing the receiver operating characteristic curve and area under receiver operating characteristic curve (AUROC) score, precision‐recall curve and area under precision‐recall curves (AUPRC) score, true positive rate (TPR), false positive rate (FPR), and probability (P) threshold, for the Ser/Thr model, and similarly **(F‐H)** for the Tyr model.

All the qPTMplants phosphoprotein sequences were used as an input for the ProtT5‐XL‐U50 pre‐trained pLM (Elnaggar et al., 2021) that was selected based on the improved performance when used at protein classification tasks compared to other pre‐trained pLMs (Zuallaert et al., 2022). From the encoded embedding vectors for each phosphoprotein, we extracted the Ser, Thr, and Tyr residue embedding vectors and the protein‐wise average embedding vector (Figure 1a). PhosBoost was designed as a machine‐learning stacking classifier that trains two separate binary classification models, one for Ser/Thr and one for Tyr prediction, using both the residue and protein embedding vector input data (Figure 1b). The stacking classifier consists of two CatBoost base classifiers, one classifier using balanced class weights based on label frequency and one CatBoost base classifier that uses equal class weights for the positive and negative labels (Figure 1b). The predicted probability results from the two stacked base classifiers were used as input features for the logistic regression metaclassifier that was trained using a 5‐fold cross‐validated prediction approach (Figure 1b).

### 2.3 Assessing the performance of the stacking classifier

The performance of the PhosBoost stacking classifier was compared to the independently trained CatBoost classifiers, one trained with balanced class weights and one with equal class weights, trained and evaluated on the same *A. thaliana* qPTMplants dataset. Considering the S/T model, all three classifiers had a similar area under the receiver operating characteristic (AUROC) score of .88 (Figure 1c), while the balanced class weights CatBoost classifier had a lower area under precision‐recall (AUPRC) score of .48 compared to .53 and .54 for the equal class weights CatBoost classifier and PhosBoost, respectively (Figure 1d). PhosBoost achieved a higher recall score at all probability thresholds (Figure 1e). A similar pattern was observed for the Y‐model binary classifiers. While all models had similar AUROC scores of .70, .71, and .72 for PhosBoost, balanced class weights CatBoost classifier and equal class weights CatBoost classifier, respectively (Figure 1f), the balanced class weights CatBoost classifier had a lower AUPRC score of .48 compared to .53 and .54 for the equal class weights CatBoost classifier and PhosBoost, respectively (Figure 1g). PhosBoost achieved a higher recall score at all probability thresholds (Figure 1h). To provide additional support for using the PhosBoost stacking classifier approach, we conducted a similar analysis on an independent dataset, the Ramasamy22 protein phosphorylation dataset, obtained from the PhosphoLingo preprint (Zuallaert et al., 2022). As with the results obtained for the data trained on *A. thaliana* qPTMplants dataset, we observed that the PhosBoost stacking classifier provided an increased recall with no cost to precision for both the S/T and Y models (Figure S3).

### 2.4 Evaluation of the predictive performance of PhosBoost in comparison to established protein phosphorylation prediction methods

**FIGURE 2:**
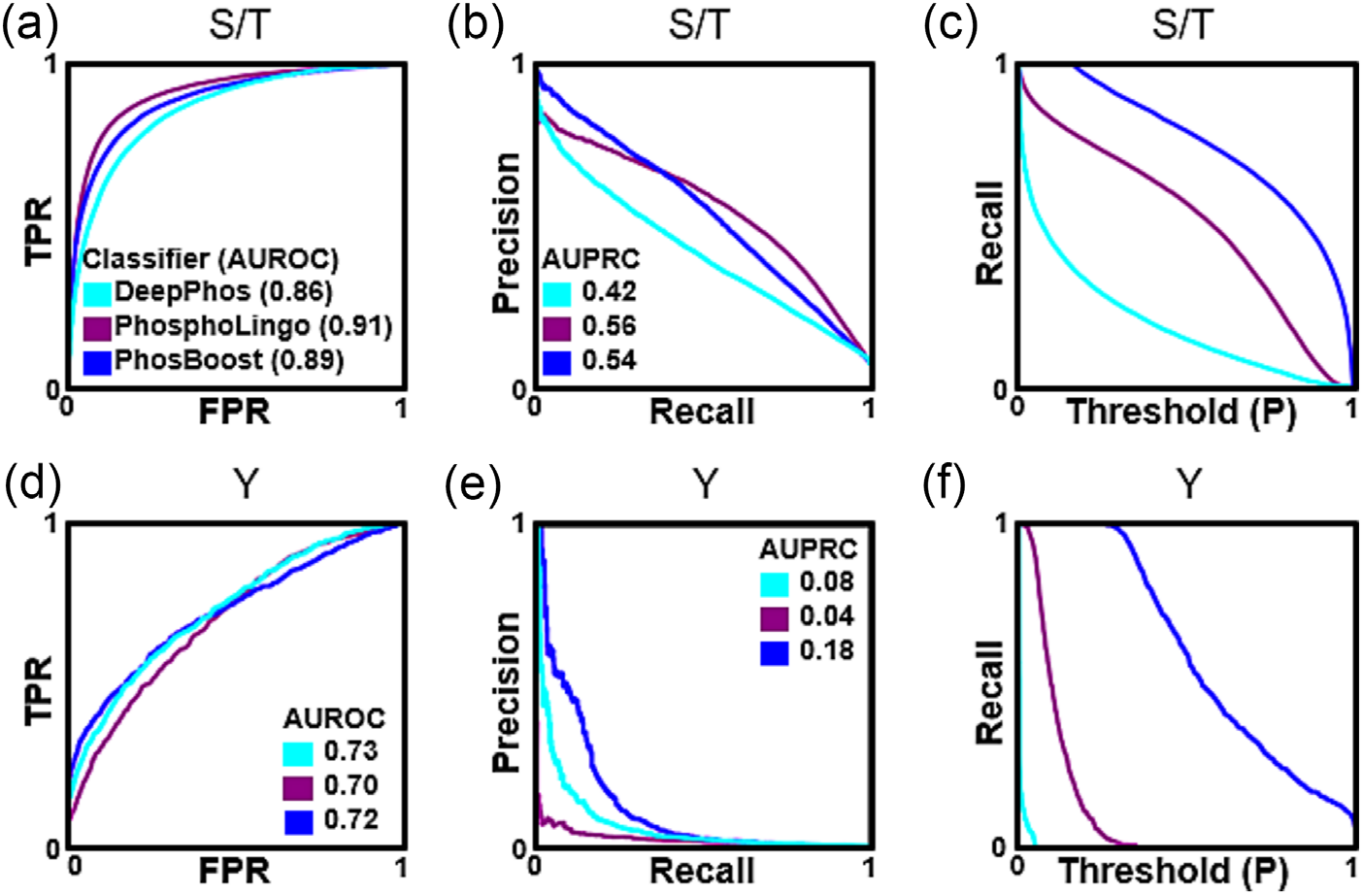
Comparing the predictive performance of PhosBoost, PhosphoLingo, and DeepPhos. (a‐c) Comparison of the performance results for DeepPhos (teal), PhosphoLingo (purple), and PhosBoost (blue), showing the receiver operating characteristic curve and area under receiver operating characteristic curve (AUROC) score, precision‐recall curve, and area under precision‐recall curves (AUPRC) score, true positive rate (TPR), false positive rate (FPR), and probability (P) threshold, for the Ser/Thr model, and similarly (d‐f) for the Tyr model.

We compared the performance of PhosBoost with two other existing protein phosphorylation classifiers, namely DeepPhos and PhosphoLingo (Luo et al., 2019; Zuallaert et al., 2022). The three methods were trained and evaluated on the same *A. thaliana* qPTMplants dataset. Considering the S/T model, PhosBoost performed better than DeepPhos but just under PhosphoLingo based on the AUROC scores (.86, .91, and, .89, respectively) (Figure 2a, Table 1) and AUPRC scores .54, .56, and .42, respectively, but lower than PhosphoLingo based on the F1 score, .43, .56, and .24, respectively (Figure 2b, Table 1). PhosBoost only achieved a higher precision score than PhosphoLingo when recall was below .41 (Figure 2b). PhosBoost achieved a higher recall score than PhosphoLingo and DeepPhos, .78, .56, and .14, respectively, and higher recall at all probability thresholds (Figure 2c, Table 1). Considering the Y model, we observed a similar pattern on the AUROC scores as for the S/T model, with PhosBoost having a higher score than DeepPhos but lower than PhosphoLingo, .72, .7, and .73, respectively (Figure 2d. Table 1). Unlike the S/T model, PhosBoost performed better than PhosphoLingo and DeepPhos based on the AUPRC scores, .14, .08, and .04, respectively, and based on the F1 score, .06, 0, and 0, respectively (Figure 2e, Table 1). Similar to the S/T model, PhosBoost achieved higher recall at all probability thresholds compared to DeepPhos and PhosphoLingo, and while the recall score for PhosBoost was .6, both DeepPhos and PhosphoLingo did not predict any Tyr phosphosites correctly (Figure 2f, Table 1).

**TABLE 1:** Comparing the classification metrics of PhosBoost with other existing phosphorylation prediction methods. The different protein phosphorylation methods were compared using different metrics, including the area under the receiver operating characteristic curve (AUROC), precision, recall, the area under the precision‐recall curve (AUPRC), and F1 scores. The scores are presented for both the S/T and Y binary classifiers.

	AUROC		Precision		Recall		AUPRC		F1	
Method	S/T	Y	S/T	Y	S/T	Y	S/T	Y	S/T	Y
DeepPhos	.86	**.73**	**.67**	0	.14	0	.42	.08	.24	0
PhosphoLingo	**.91**	.70	.57	0	.56	0	**.56**	.04	**.56**	0
PhosBoost	.89	.72	.29	**0.03**	**.78**	**.60**	.54	**.14**	.43	**.06**

Because neither PhosphoLingo nor DeepPhos correctly predicted Tyr phosphosites in the *A. thaliana* dataset, we decided to conduct an additional benchmark using a different dataset, namely the Ramasamy22 human phosphoproteomic dataset obtained from the PhosphoLingo preprint (Zuallaert et al., 2022). To compare the label imbalance between the two datasets, we analyzed the number of unique positive and negative phosphosites. For the negative phosphosites, we found that there were approximately twice as many Ser, Thr, and Tyr phosphosites in the *A. thaliana* qPTMplants dataset (Figure S2A). For the positive phosphosites, there were approximately twice as many Ser and Thr phosphosites in the *A. thaliana* qPTMplants dataset but approximately half the number of Tyr phosphosites (Figure S2B), explaining the observed similar label imbalance for the Ser and Thr phosphosites but higher Tyr label imbalance in the *A. thaliana* qPTMplants dataset (Figure S2C). Considering the S/T model, PhosBoost had a lower AUROC score than DeepPhos and PhosphoLingo, .90, .92, and .94, respectively (Figure S4A). Based on the AUPRC score, PhosBoost performed worse than PhosphoLingo but better than DeepPhos, .63, .71, and .51, respectively (Figure S4B). PhosBoost achieved a higher recall score of .83, compared to .64 and .26 for PhosphoLingo and DeepPhos, respectively, while achieving a higher recall score at all probability thresholds (Figure S4C). Considering the Y model, PhosBoost had a higher AUROC score than PhosphoLingo and DeepPhos, .93, .75, and .9, respectively (Figure S4D) and a higher AUPRC score, .55, .45, and .10, respectively (Figure S4E). PhosBoost achieved a higher recall score of .74, compared to .31 and .00 for PhosphoLingo and DeepPhos, respectively, while achieving a higher recall score at all probability thresholds (Figure S4F).

### 2.5 Analysis of the predicted positive phosphosites suggests that PhosBoost provides more confident scores and outperforms PhosphoLingo and DeepPhos if recall is prioritized

**FIGURE 3:**
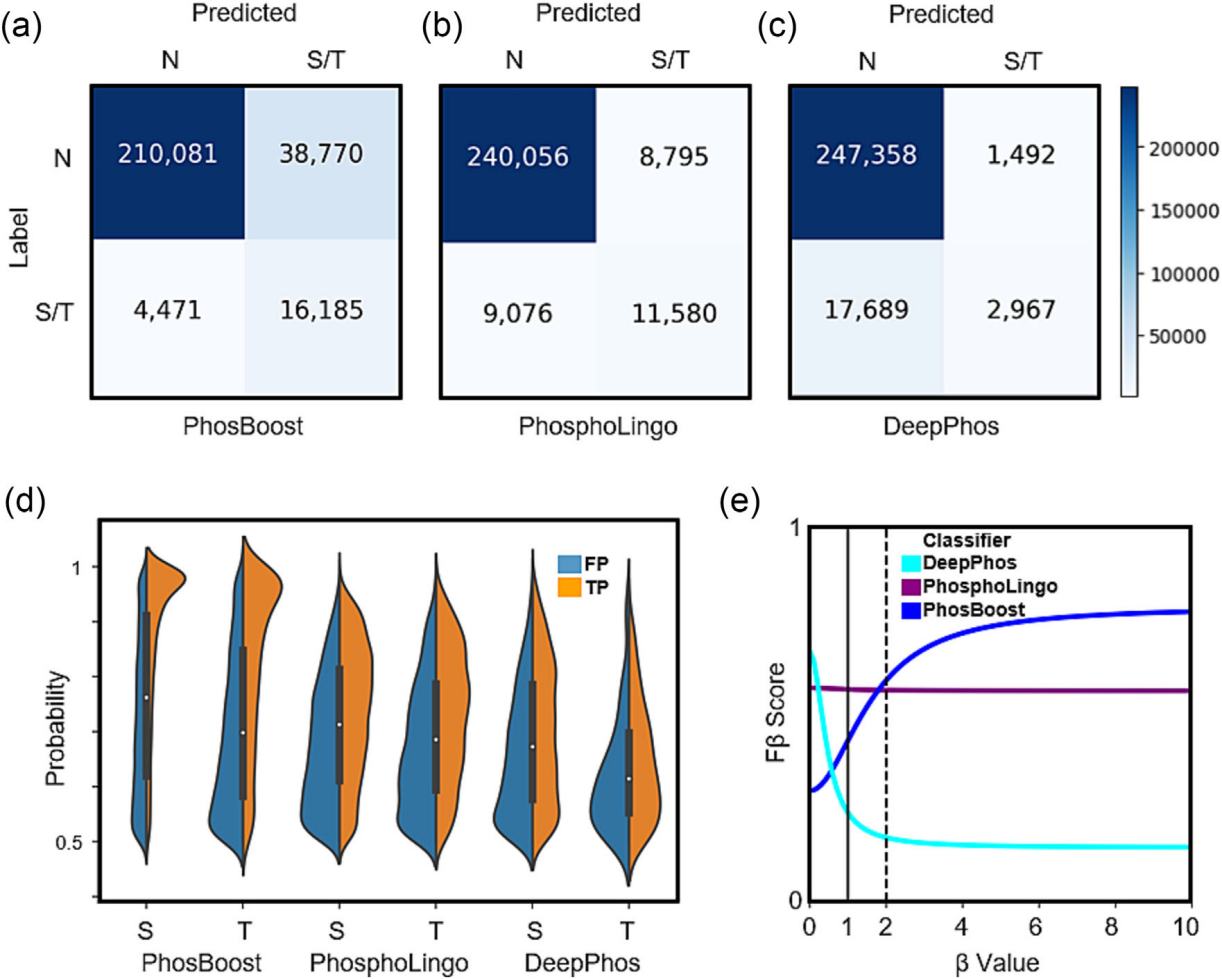
Despite lower precision, the PhosBoost S/T model produces more informative predicted probability scores and achieves better performance when recall is prioritized. (a‐c) confusion matrices for the S/T model results for PhosBoost, PhosphoLingo, and DeepPhos, respectively (N stands for negative phosphosites). (d) A split violin plot showing the distribution of the predicted probability values for all true positive (TP) and false positive (FP) samples (predicted probability > .5) separated by serines (S) and threonines (T), for PhosBoost, PhosphoLingo, and DeepPhos, respectively. (E) Evaluation of the PhosBoost, PhosphoLingo, and DeepPhos model performances using the Fβ measure at different β values. All results are based on models trained on the *A. thaliana* qPTMplants dataset. The heatmap legend shown is shared across the confusion matrices.

While achieving higher performance at Tyr phosphosite prediction (Figure 2d‐e, S4D‐E, Table 1), PhosBoost performance at Ser/Thr prediction was better than DeepPhos but lower or comparable to PhosphoLingo (Figure 2b, S4B, Table 1). Despite the differences in performance, PhosBoost consistently obtained higher recall scores than PhosphoLingo and DeepPhos (Figure 2c, f, S4C, S4F, Table 1), pointing to a potential tradeoff between precision and recall. First, we plotted the confusion matrices to compare the results of the S/T models on the *A. thaliana* qPTMplants test set. The results show that the number of true positive (TP) Ser/Thr phosphosites predicted by PhosBoost, PhosphoLingo, and DeepPhos were 16,185, 11,580, and 2,967, respectively, and the number of false positives (FP) was 38,770, 8,795, and 1,492, respectively (Figure 3a‐c). Focusing on the PhosBoost Y model results, due to the lack of correctly predicted Y phosphosites by DeepPhos and PhosphoLingo (Figure 2f, Table 1), the results show that 12,448 and 391 Tyr phosphosites were predicted as FP and TP, respectively (Figure S5A). Previously, we observed that PhosBoost had higher precision than PhosphoLingo at lower recall (Figure 2b), suggesting a difference in the distribution of the predicted probability scores. The split violin plots show the distribution of the TP and FP predicted phosphosites by PhosBoost, PhosphoLingo, and DeepPhos, for both the Ser and Thr phosphosites (Figure 3d). We observed that for all three classifiers, the predicted probability scores of the TPs were generally higher than of the FPs. The difference between the distributions was most distinct for PhosBoost (Figure 3d). Notably, the peak of the distribution for the TP predicted probability scores, for both the Ser and Thr results, was between .9 and 1 (Figure 3d). In contrast to the Ser and Thr results, we observed a bi‐modal distribution for the true positive Tyr results, with one peak between .8 and 1 and another peak between .5 and .7 (Figure S5B). A similar pattern to the S/T results was observed for the S/T model trained on the Ramsamy22 dataset (Figure S6A‐C) and the Y model (Figure 6d‐f), although the distribution of predicted probability values for PhosBoost and PhosphoLingo were similar for the S/T results, with TPs having a peak approximate between .9 and 1.0 (Figure S6G), the TP Tyr phosphosites having a peak approximate between .9 and 1.0 only for PhosBoost (Figure S6G).

While the F1 score assumes equal importance to the precision and recall, depending on the stated objective for the classification method, it is possible to assess the precision‐recall tradeoff using a weighted Fβ score. The generalized Fβ‐score produces an F‐score for different β values that evaluate the classifier performance under the assumption that recall is β‐times as important as precision. To compare the performance of PhosBoost, PhosphoLingo, and DeepPhos, we plotted the Fβ score over different β values for the *A. thaliana* qPTMplants results. Based on this plot we showed that as the β increases, the Fβ score for PhosBoost increases, remains relatively uniform for PhosphoLingo, and decreases for DeepPhos (Figure 3e). A similar observation was made for the Tyr results, whereas the β increases, the Fβ score for PhosBoost increases (Figure S5C). Furthermore, while the F1 score of PhosphoLingo is higher than PhosBoost and DeepPhos (Table 1), when the β is equal to 1.8 the Fβ score of PhosBoost reaches the Fβ‐score of PhosphoLingo, suggesting that if recall is considered to be approximately twice as important as precision, PhosBoost performs better than PhosphoLingo and DeepPhos (Figure 3e). Similar results were obtained when comparing the performance of PhosBoost, PhosphoLingo, and DeepPhos using the Fβ score at different β values on the Ramsamy22 dataset, but with a slightly higher β value of 2 for the S/T model (Figure S6H) and a lower β value of 1.6 for the Y model (Figure S6I).

### 2.6 A DIAMOND pairwise sequence alignment‐based approach can be used to supplement protein phosphorylation prediction methods to improve phosphosite prediction and annotation

**FIGURE 4:**
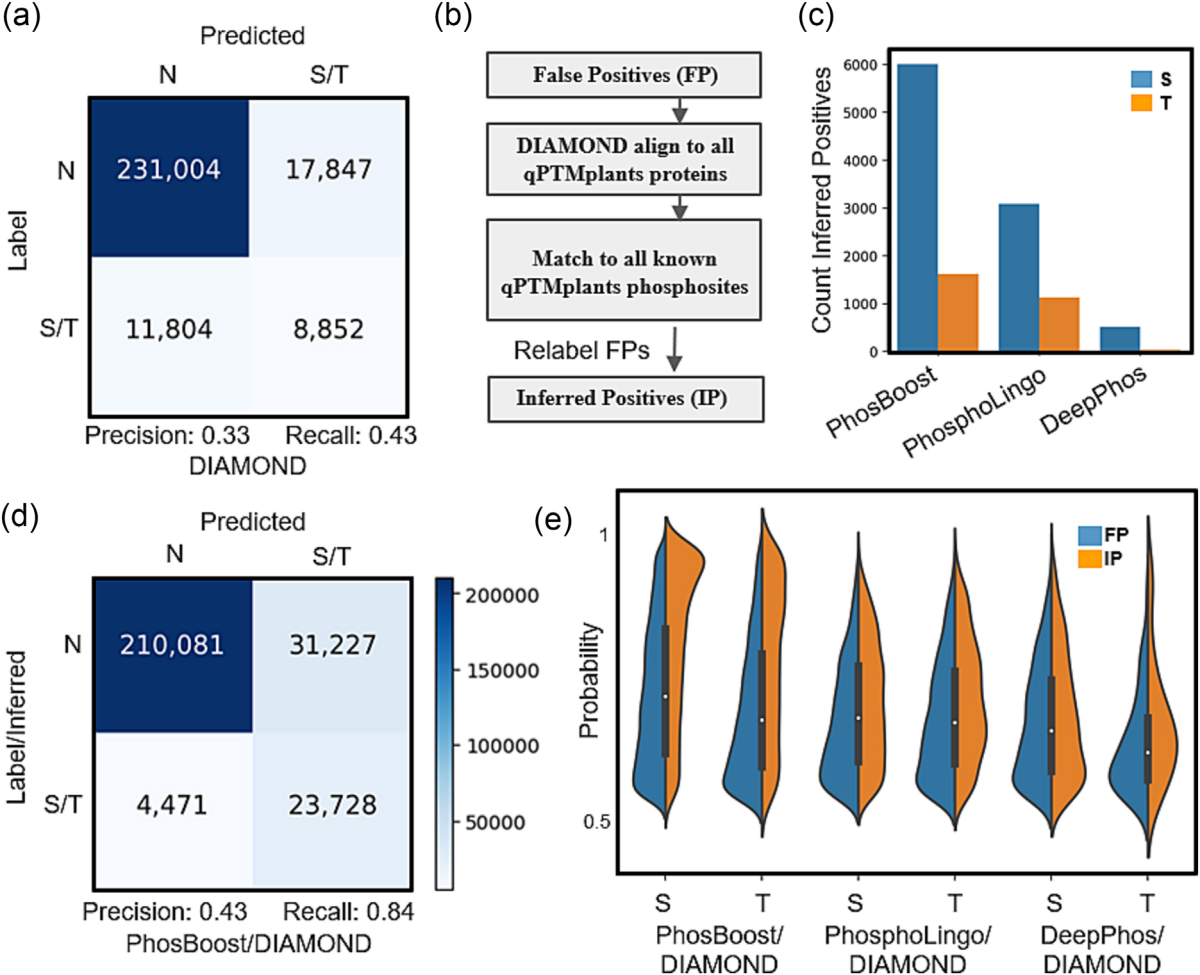
Using a DIAMOND‐based pairwise alignment analysis improves phosphosite annotation and reduces false positive label uncertainty. (a) A confusion matrix using a DIAMOND‐based binary protein phosphorylation prediction was trained and tested on the *A. thaliana* qPTMplants dataset. (b) A schema representing the workflow that uses DIAMOND to evaluate false positive (FP) sites to identify inferred positive (IP) sites. (c) A bar graph showing the number of IP serines (S) and threonines (T) inferred from the PhosBoost, PhosphoLingo, and DeepPhos FP results. (d) Confusion matrix for the PhosBoost S/T model results after combining true positive and IP phosphosites accounting for the FP phosphosites. (e) A split violin plot showing the distribution of the predicted probability values for all FP and IP phosphosites (predicted probability > .5) for the PhosBoost, PhosphoLingo, and DeepPhos results, separated by serines (S) and threonines (T). In all confusion matrices, N stands for non‐phosphorylated. The heatmap legend shown is shared across the confusion matrices.

Sequence alignment‐based approaches were shown to be useful for phosphosite annotation and prediction by searching for similarity between sequences surrounding phosphosites and experimentally derived protein phosphorylation data (Chaudhuri et al., 2015; Maathuis, 2008; Tan et al., 2009). Due to the higher number of predicted false positives by PhosBoost, compared to DeepPhos and PhosphoLingo, we considered using a DIAMOND protein pairwise alignment‐based approach to both evaluate the predicted false positives and improve phosphosite annotation (Buchfink et al., 2021). We first assessed the performance of such an approach when used as a protein phosphorylation prediction method on the *A. thaliana* qPTMplants data. Using DIAMOND, we extracted peptide sequences of size 31 centered at the Ser/Thr phosphosites in the test dataset and conducted pairwise alignments to identify matching experimental phosphosites in the training and validation datasets. We were able to correctly classify 8,852 of the 20,656 experimentally derived Ser and Thr phosphosites, achieving a precision score of .33 and a recall score of .43 (Figure 4a). Furthermore, we found that 17,847 Ser/Thr negative phosphosites matched experimentally derived phosphosites (Figure 4a). For the experimentally derived Tyr phosphosites, we were able to correctly classify 168 of the 654 sites, achieving a precision score of .06 and recall score of .26, and found 2,444 Tyr negative phosphosites that matched experimentally derived phosphosites (Figure S7A).

Next, we used the DIAMOND‐based pairwise alignment step to estimate the improvement of the phosphosite annotation, focusing on the predicted false positive sites. We aligned all false positive Ser and Thr phosphosites predicted by PhosBoost, PhosphoLingo, and DeepPhos to the complete qPTMplants database. Phosphosites matching any experimentally derived phosphosite, excluding self‐matches, were relabeled as inferred positives (IP) and annotated with the supporting information (Figure 4b). Based on this analysis, we were able to relabel 7,543 FP Ser and Thr phosphosites predicted by PhosBoost, 4,082 by PhosphoLingo, and 482 by DeepPhos (Figure 4c). Thus, in the case of PhosBoost, by combining the inferred positives with the true positive labels, we were able to increase the number of predicted and inferred true positive Ser and Thr phosphosites from 16,185 to 23,728 and reduce the number of false positives from 38,770 to 31,227, while achieving a precision score of .43 and a recall score of .84 (Figure 4d). For the Tyr results, we were able to increase the number of predicted and inferred true positive phosphosites from 391 to 1,375 and reduce the number of false positives from 12,448 to 11,464, while achieving a precision score of .11 and a recall score of .84 (Figure S5A, S7B). We also assessed whether the predicted probability scores for the inferred positive Ser and Thr phosphosites differ from the false positives in PhosBoost, PhosphoLingo, and DeepPhos. When comparing the distribution of the probability scores between the false positives and inferred positives, we observed that the largest difference between the probability distributions, for both Ser and Thr phosphosites, was for PhosBoost, with a distribution peak approximately between probability scores of .9 and 1.0, while the distribution for the predicted probability scores for the false positives and inferred positives were relatively similar for PhosphoLingo and DeepPhos (Figure 4e). In contrast, for the Tyr results, we found that the distribution peaks for both false positives and inferred positives are approximately between probability scores of .5 and .7 (Figure S7C).

### 2.7 Assessing the transferability of models trained on *A. thaliana* data at predicting protein phosphorylation in other plant species

**FIGURE 5:**
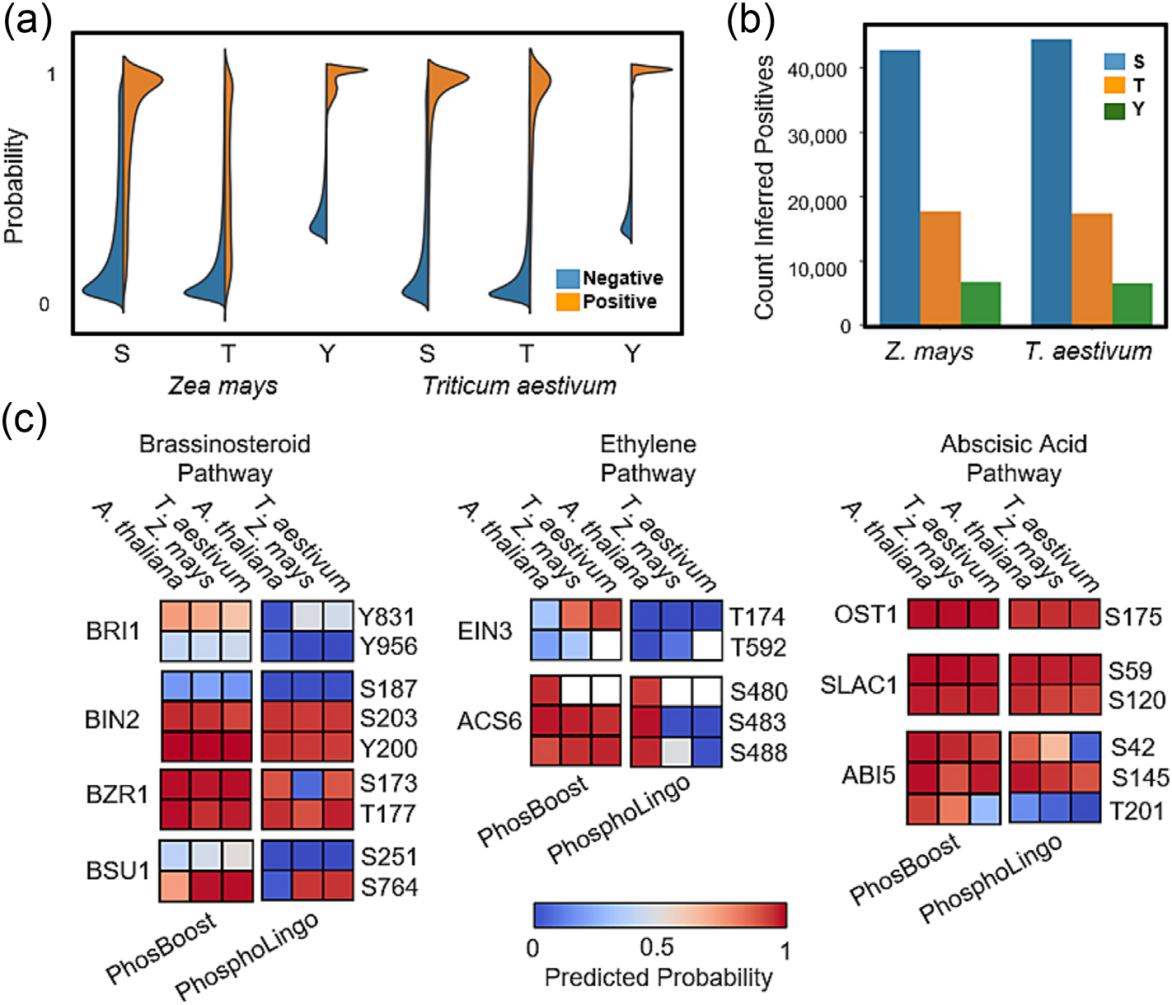
PhosBoost protein phosphorylation predictions are transferable across plant species and perform better than PhosphoLingo on a number of functionally important phosphosites. (a) A split violin plot showing the distribution of the predicted probability scores for all phosphosites present in the *Zea mays* and *Triticum aestivum* qPTMplants database predicted using the S/T and Y PhosBoost models trained on the *A. thaliana* qPTMplants dataset. The results show the probability scores for all negative phosphosites (blue) and positive phosphosites (orange) separated by serines (S), threonines (T), and tyrosines (Y) phosphosites. (b) A bar graph showing the number of inferred positive (IP) S, T, and Y phosphosites as detected by using DIAMOND to align the predicted false positive phosphosites by the S/T and Y PhosBoost models to the complete qPTMplants database. (c) Analysis of the predicted probability scores for PhosBoost and PhosphoLingo on a small number of verified functional *A. thaliana* phosphosites in the brassinosteroid, ethylene, and abscisic acid phytohormone pathways and the matching phosphosites in the top blast hit in *Z. mays* and *T. aestivum* . Non‐conserved sites are filled with white background.

We wanted to assess the ability of the S/T and Y PhosBoost models trained on the *A. thaliana* qPTMplants dataset to correctly predict known functional phosphosites in more distantly related plant species such as *Z. mays* and *T. aestivum*. Compared to the available experimental protein phosphorylation for *A. thaliana* in the qPTMplants database, a much smaller amount of data is available for other plant species, including *Z. mays* and *T. aestivum* (Figure S1A). The resulting lack of phosphosite saturation and higher label imbalance in *Z. mays* and *T. aestivum*, compared to *A. thaliana*, suggests that the use of standard metrics, such as precision, recall, AUPRC, and F1 scores, to assess the performance of protein phosphorylation models are not as informative. Therefore, to assess the transferability of models trained on *A. thaliana* data, we used split violin plots to show the distributions of the probability score predicted by the S/T and Y PhosBoost models, across the positive and negative Ser, Thr, and Tyr phosphosites (Figure 5a). The observed difference in the predicted probability scores for the positive and negative Ser, Thr, and Tyr shows that in general, positive phosphosites were predicted with a higher probability score than negative ones, with a peak distribution approximately between .9 and 1, albeit a smaller peak for Thr predictions (Figure 5a). Furthermore, we detected over 40,000, close to 20,000, and close to 10,000 inferred positive Ser, Thr, and Tyr phosphosites, respectively, in both *Z. mays* and *T. aestivum* (Figure 5b).

For a concise prediction comparison, we compiled a short list of functionally important phosphosites, validated in *A. thaliana*, involved in the brassinosteroid (BR), ethylene (ET), and abscisic acid (ABA) pathways. In the BR pathway, we included BRASSINOSTEROID INSENSITIVE 1 (BRI1) Y831 and Y956 (Bojar et al., 2014; Oh et al., 2009), BRASSINOSTEROID‐INSENSITIVE2 (BIN2) S187, S203, and Y200 (Kim et al., 2009; Xiong et al., 2017), BRASSINAZOLE RESISTANT1 (BZR1) Thr173 and Thr177 (Ryu et al., 2007), and BRI1‐SUPPRESSOR1 (BSU1) Ser251 and Ser764 (Park et al., 2022). In the ET pathway, we included ETHYLENE INSENSITIVE3 (EIN3) Thr174 and Thr592 (Zhao & Guo, 2011) and ACC SYNTHASE 6 Ser480, Ser483, and Ser488 (Liu & Zhang, 2004). In the ABA pathway, we included OPEN STOMATA1 (OST1) Ser175 (Belin et al., 2006), SLOW ANION CHANNEL‐ASSOCIATED1 (SLAC1) Ser59 and Ser120 (Brandt et al., 2015), and ABSCISIC ACID INSENSITIVE5 (ABI5) Ser42, Ser145, and Thr201 (Y. Wang et al., 2013). The top blast hit in *Z. mays* and *T. aestivum* for each of the *A. thaliana* protein sequences was used to identify the reciprocal phosphosite. We then used PhosBoost and PhosphoLingo, trained on the *A. thaliana* qPTMplants dataset to generate the predicted probability scores for each phosphosite (Figure 5c, Table S2). The prediction results show that except for BRI1‐Y956, BIN2‐S187, BSU1‐S251, and EIN3‐T174/T592 for PhosBoost and PhosphoLingo, and BRI1‐Y831, BSU1‐S764, and ABI5‐T201 for PhosphoLingo, most of *the A. thaliana* sites were correctly predicted by both methods (Figure 5c). For the *Z. mays* and *T. aestivum*, PhosBoost correctly predicted more of the phosphosites than PhosphoLingo and with a higher predicted probability score for most phosphosites (Figure 5c). PhosBoost also generated more consistent predictions across the three species tested, with the exception of EIN3‐T174 and ABI5‐T201 for PhosBoost and PhosphoLingo, and BZR1‐S173, BSU1‐S764, ACS6‐S483/S488, and ABI5‐S42/T201 (Figure 5c).

### 2.8 Using PhosBoost for genome‐wide protein phosphorylation predictions and integration within genome browsers

**FIGURE 6:**
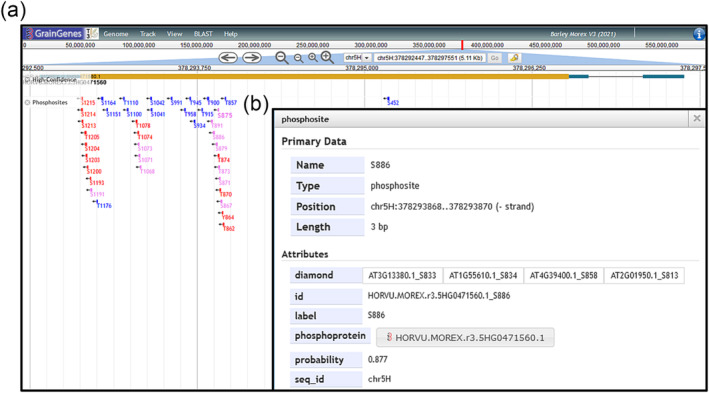
Integrating the PhosBoost prediction results as a track within the JBrowse genome browser for better accessibility. For this example, we generated a GFF3 file that contains all positive and inferred predicted phosphosites by PhosBoost for all protein sequences in the *H. vulgare* Morex version 3 genome assembly, using one representative protein sequence for each gene model. All phosphosites were mapped to their respective 3 bp genomic coordinates. (a) Screenshot of the PhosBoost predicted phosphosites track view for the gene HORVU.MOREX.r3.5HG0471560.1. In this example, phosphosites were color‐coded as follows: blue for phosphosites inferred by DIAMOND pairwise sequence alignment, red for phosphosites with predicted probability scores above .5, and pink if both cases apply. The labels of phosphosites with predicted probability scores above .9 are shown in bold font. (b) A view of one of the PhosBoost predicted phosphosites, showing the phosphosite ID, predicted probability score, and the ID of the DIAMOND pairwise sequence alignment matches with experimentally derived phosphosites in the qPTMplants database.

In this study, we aimed to use PhosBoost as a scalable machine‐learning method for generating genome‐wide protein phosphorylation predictions. For this, we trained new S/T and Y PhosBoost models on the complete qPTMplants datasets to be used for plant phosphosite predictions. We then used PhosBoost to conduct genome‐wide protein phosphorylation predictions in four plant species (one accession per species): wheat (Chinese Spring), oat (Sang), barley (Morex), and maize (B73), using one representative protein sequence for each gene model. Additionally, the DIAMOND pairwise alignment analysis was used to improve the annotation of all phosphosites using the complete qPTMplants dataset as a reference. To achieve this, we developed a straightforward approach that converts the prediction and annotation results into a GFF3 format, allowing for direct integration within JBrowse genome browsers (Figure 6a) (Diesh et al., 2023). In this example, the labels of phosphosites with predicted probability above .9 were marked in bold font, and labels were color‐coded as follows: blue for phosphosites inferred by DIAMOND pairwise sequence alignment, red for phosphosites with predicted probability scores above .5, pink if both cases apply (Figure 6a). Each phosphosite contains additional metadata such as the phosphosite ID, predicted probability score and DIAMOND pairwise sequence alignment matches (Figure 6b). These results are now accessible through the GrainGenes (Yao et al., 2022) and MaizeGDB databases (Woodhouse et al., 2021).

## 3 DISCUSSION

Protein phosphorylation is an important and dynamic post‐translational modification that has a wide‐spread effect on many biological processes by regulating protein functions and interactions (Nishi et al., 2014). Compared to *A. thaliana*, experimental phosphorylation data for most other plant species are either not available or are relatively small and would require additional high‐throughput phosphoproteomic experiments to achieve a similar degree of saturation (Figure S1A‐C). Therefore, the prediction of protein phosphosites is instrumental in elucidating possible regulatory phosphosites where extensive experimental phosphoproteomic data is not available (Meng et al., 2022). While the use of state‐of‐the‐art deep learning and pLMs for protein phosphorylation prediction improved performance over existing methods (Zuallaert et al., 2022), classical machine learning approaches, such as gradient boosting trees, remain as powerful and scalable classification methods (Anghel et al., 2019; Lyashevska et al., 2021). Furthermore, ensemble methods that combine different machine‐learning classification methods can improve classification performance by learning from multiple weak classifiers (Dietterich, 1997). Choosing appropriate class weights during training can have a substantial effect on the performance of the trained machine‐learning classification model, including the popular use of balanced class weights for highly imbalanced data (Cui et al., 2019; Liu & Zhou, 2006). In designing PhosBoost, we observed that a stacking classifier consisting of two CatBoost base classifiers, one trained with balanced class weights and one trained with equal class weights, outperforms both independently trained CatBoost classifiers on two different datasets, providing an increased recall with no cost to precision for both the S/T and Y models (Figure 1c‐h, Figure S3A‐F). We hypothesize that the use of balanced class weights to train one base classifier helps address the class imbalance by giving more weight to the underrepresented positive class while the base classifier trained with equal class weights captures the overall characteristics of the dataset, effectively learning from the majority negative class, allowing the stacking classifier to synergistically learn from these complementary approaches.

Next, we benchmarked the performance of PhosBoost and two existing protein phosphorylation prediction methods, namely PhosphoLingo and DeepPhos, on two separate datasets. For the S/T model, we observed that the performance of PhosBoost, based on the AUPRC scores, was comparable to PhosphoLingo on the *A. thaliana* qPTMplants, lower on the Ramasamy22 data, and higher than DeepPhos on both datasets (Figure 2b, S4B, Table 1). On the other hand, for the Y model, PhosBoost performed better than PhosphoLingo and DeepPhos on both datasets (Figure 2e, S4E, Table 1). Different data‐centric factors, such as dataset size and label imbalance, can have a substantial impact on model performance (L. Dou et al., 2021; Lyashevska et al., 2021). A comparison of the S/T and Y model performances in relation to data size and imbalance, suggests that data size impacted the S/T model performance and data imbalance impacted the Y model performance when comparing the models trained on the *A. thaliana* qPTMplants and Ramasamy22 datasets (Figure S2A‐C, 2a‐f, S4A‐F). Based on our results, we hypothesize that the performance of PhosBoost improves when the input data increases in size, as observed for the S/T model (Figure S2A‐B), or increases in label imbalance, as observed for the Y model (Figure S2C). We also demonstrate that PhosBoost consistently achieves higher recall scores at all probability thresholds compared to PhosphoLingo and DeepPhos (Figure 2c, f, S4C, S4F). Thus, we show that PhosBoost can be competitive with existing protein phosphorylation prediction methods, particularly when higher recall and improved predicted phosphosite coverage are beneficial.

Despite the improved recall score, PhosBoost presents a precision‐recall tradeoff associated with an increased number of predicted false positives compared to PhosphoLingo and DeepPhos (Figure 3a‐c, Figure S5A). We assessed the performance of PhosBoost, PhosphoLingo, and DeepPhos under the assumption that, depending on the aim of the prediction method, the importance of recall can outweigh precision. We show that PhosBoost has a higher Fβ score when the value of the β parameter is approximately 2 for both the *A. thaliana* qPTMplants and Ramsamy22 datasets, in the S/T and Y models (Figure 3d, S6H‐I). Therefore, in view of the precision‐recall tradeoff, we suggest that when a recall is prioritized and considered to be twice as important as precision, PhosBoost achieves higher performance than PhosphoLingo and DeepPhos on both the *A. thaliana* qPTMplants and Ramasamy22 datasets. Furthermore, we show that PhosBoost consistently produces predicted probability scores that are more indicative of whether a phosphosite are true or false positive, providing a more confident and informative predicted probability score (Figure 3d, S6G). Taken together, our results suggest that PhosBoost achieves higher performance than PhosphoLingo and DeepPhos when recall is prioritized over precision while providing more confidence through more informative predicted probability scores.

Due to complex phosphorylation and dephosphorylation dynamics and technical limitations, many phosphosites remain undetected by high throughput phosphoproteomic approaches (Doll & Burlingame, 2015; Gelens & Saurin, 2018; Iakoucheva, 2004). One of the approaches used to infer if a phosphosite is positive despite the lack of experimental data is through the identification of conserved phosphosites with experimental evidence (Chaudhuri et al., 2015). To partially address the problem of negative phosphosite label uncertainty, we have developed a supplementary pairwise sequence alignment analysis step using DIAMOND to improve phosphosite annotation. We show that this approach can be used by any protein phosphorylation classification method to increase the number of inferred positive phosphosites (Figure 4c), reduce the number of false positives (Figure 4d), and provide useful information about the matching experimental phosphosites. Furthermore, by comparing the probability score distribution of the false and inferred positive phosphosites, we show that PhosBoost produces higher predicted probability scores for inferred positives than false positives for Ser and Thr phosphosites, providing a more confident and informative predicted probability score than PhosphoLingo and DeepPhos (Figure 4e). For Tyr phosphosites, PhosBoost was not as informative at predicting inferred positives compared to false positives (Figure S7C), possibly due to higher label imbalance (Figure S2C). Label uncertainty and mislabeling is a common problem for protein phosphorylation classification methods that could be partially addressed by improving phosphosite annotation prior to training and testing, with a possible benefit of improved model performance and interoperability.

One of our goals when developing PhosBoost was to develop a method that is scalable for genome‐wide protein phosphorylation prediction. Because a large portion of the experimental protein phosphorylation data in the qPTMplants belongs to *A. thaliana* phosphosites, we assessed whether PhosBoost trained on the *A. thaliana* dataset can effectively predict phosphosites in other plant species. Comparison of the distribution of the predicted probability scores for Ser, Thr, and Tyr, provides compelling evidence that the S/T and Y models can provide informative predicted probability scores that effectively distinguish between true and false positive phosphosites. We also show that the DIAMOND pairwise alignment step can be used to improve the phosphosite annotation of a large number of Ser, Thr, and Tyr phosphosites, in both *Z. Mays* and *T. aestivum*. A comparative analysis of PhosBoost and PhosphoLingo on a small number of known functional phosphosites from *A. thaliana* with matching phosphosites from *Z. mays* and *T. aestivum* showed that PhosBoost correctly predicted a larger number of phosphosites with higher predicted probability scores than PhosphoLingo. Based on these results, we trained final the final PhosBoost S/T and Y models on the complete qPTMplants protein phosphorylation dataset and conducted genome‐wide protein phosphorylation prediction on four plant species, *Z. mays*, *T. aestivum*, *Avena sativa*, and *Hordeum vulgare*. To facilitate the accessibility to the prediction data, we developed a method that converts the prediction results and the pairwise DIAMOND alignment results into a file that can be directly incorporated and visualized on the genome browser (Figure 6a‐b).

## 4 CONCLUSION

We present PhosBoost, a general protein phosphorylation prediction method that uses pLMs and a stacking classifier composed of CatBoost gradient boosting tree base classifiers. We show evidence to suggest that PhosBoost has improved performance when working with large and imbalanced datasets, showing comparable results for Ser/Thr classification and improved Tyr classification. We show that PhosBoost consistently achieves higher recall and more informative predicted probability scores, making PhosBoost particularly useful when recall is prioritized over precision and a higher coverage of predicted phosphosites is beneficial. We developed a DIAMOND pairwise sequence alignment analysis step to reduce phosphosite label uncertainty and improve phosphosite annotation. We show that PhosBoost is scalable to genome‐wide protein phosphorylation predictions and implement a straightforward method for integrating prediction and annotation results directly in the genome browser to facilitate accessibility.

## 5 MATERIALS AND METHODS

### 5.1 Data sources and generation of input embedding data

The complete list of phosphorylated residues from the qPTMplants database for protein post‐translational modification was used (Xue et al., 2022). Because the qPTMplants database does not provide the protein sequences for the annotated phosphoproteins, protein sequences were manually obtained from genomic databases such as UniProt (The UniProt Consortium et al., 2023), Phytozome (Goodstein et al., 2012), and EnsemblPlants (Yates et al., 2022), and processed with BioPython (v.1.81) (Cock et al., 2009). Proteins that are not present in genomic databases or contain missing or non‐canonical amino‐acid sequences were discarded. The phosphorylation positions were cross‐referenced to ensure that the qPTMplants sites matched the protein sequences, and no‐matching sequences were removed. Protein sequences containing non‐canonical or missing amino acids were removed. The resulting FASTA file was used directly as the input for calculating protein and amino acid embedding vectors using the pre‐trained ProtT5‐XL‐U50 pLM (Elnaggar et al., 2021). The Python code used to generate the embeddings was obtained from the ProtTrans GitHub repository (https://github.com/agemagician/ProtTrans) and is based on the ProtT5 pLM architecture. The code was modified to specifically return the amino‐acid embedding vectors of S/T/Y residues and the protein‐wise average embedding vector (available at https://github.com/eporetsky/PhosBoost). The combined S/T/Y amino‐acid and protein‐wise average embedding data were used, without modification, as input data for the PhosBoost classifiers. Using the generated amino acid embedding vectors and the average protein embedding vectors, all sites included in the qPTMplants database were extracted as a positive phosphorylated site set, and all the remaining S/T/Y sites were extracted as a negative phosphorylation set. Initial training and evaluation on predictive classification models were conducted using the data obtained from the complete *A. thaliana* phosphorylation dataset. For the training and validation of PhosBoost, only the *A. thaliana* data from the qPTMplants database was used. The complete *A. thaliana* dataset was randomly split into stratified training, validation, and testing sets using a 60%–20%–20%, split, respectively.

### 5.2 Overview of the PhosBoost protein phosphorylation classifier

A stacking classifier is a two‐step ensemble learning method composed of an initial stack of base classifiers followed by a final meta‐classifier that integrates the results of the base classifiers. In the first step, the training data is used to separately train the base classifiers specified within the stack. In the second step, the predicted probability scores from each base classifier are used as new training data features for training the final meta‐classifier. The metaclassifier is trained using a cross‐validated prediction approach to reduce over‐fitting by not training on the same data used to train the base classifiers. Stacking classifiers have been shown to often perform better than the individual base classifiers by combining different types of classifiers that may have complementary strengths and weaknesses while reducing bias and variance. We implemented PhosBoost as a stacking classifier composed of two stacked base‐classifiers and a metaclassifier, using a 5‐fold cross‐validation approach. The stacking classifier is comprised of two CatBoost base classifiers (v1.1.1) (Dorogush et al., 2018), one trained using the balanced class weight parameter to modify class weights according to label frequencies (auto_class_weights=“balanced”), and one trained using the default equal class weights parameters. The predicted probability scores produced by each of the two base‐classifiers were then used as input training data features for the logistic regression metaclassifier, using the balanced class weight parameter. The stacking classifier and logistic‐regression metaclassifier were implemented using the Python scikit‐learn package (v1.2.1) (Pedregosa et al., 2011). The CatBoost API is compatible with the scikit‐learn architecture, enabling direct integration of the base‐classifiers within the stacking classifier. Furthermore, to provide support for the improved performance of the stacking classifier, the stacking PhosBoost classifier performance was compared to the performance of the two independently trained CatBoost classifiers outside of the stacking classifier. Hyper‐parameter tuning was conducted on the training and validation sets, using the Bayesian optimization function BayesianOptimization from the bayes_opt Python package (v1.4.2) (Nogueira, 2014). The Bayesian optimization method was used to fine‐tune the following hyper‐parameters: “n_estimators” (between 50 and 2000), “depth” (between 2 and 10), and “learning_rate” (between .05 and .5) by optimizing the F1‐score metric over 100 iterations. Hyper‐parameters optimization was conducted separately for the two independent CatBoost classifiers, one trained with equal class weights and one trained with balanced class weights, and separately for the ST and Y models. The obtained model parameters were then used by the two respective CatBoost base‐classifiers within the ST and Y model PhosBoost stacking classifiers. The “n_estimators” and “depth” hyper‐parameters were rounded to the nearest integer, as instructed by the bayes_opt Python package (v1.4.2). The obtained values for the hyper‐parameters are available (Table S1).

### 5.3 Evaluation of protein phosphorylation predictive model performances

We benchmarked the performance of PhosBoost compared to two established protein phosphorylation classification methods, namely PhosphoLingo (v0.1.0) (Zuallaert et al., 2022) and DeepPhos (Luo et al., 2019). All three classification methods produce two separate binary classifiers, the S/T and Y models. PhosBoost and PhosphoLingo used the same training and validation sets for hyperparameter tuning, while DeepPhos used the combined training and validation sets for internal hyperparameter tuning and training. For PhosphoLingo we used the pre‐trained ProtT5‐XL‐U50 pLM model to train a protein phosphorylation model under the “full” setting. The default settings were used in the training and testing process of DeepPhos.

### 5.4 Building a DIAMOND‐based inference method to improve phosphosite annotations

To enhance phosphosite annotation, we employed a sequence‐based DIAMOND alignment step (v2.1.6) (Buchfink et al., 2021). We extracted a peptide of length 31 for each candidate phosphosite of Ser, Thr, and Tyr within a protein sequence, with the phosphosite at the center of the sequence. For phosphosites positioned at the edges of protein sequences, a peptide of length 31 was extracted but with the phosphosite position being determined based on the distance from either edge of the protein sequence. After obtaining all peptide sequences, each peptide was aligned using DIAMOND (−‐masking none ‐‐ultra‐sensitive ‐‐max‐target‐seqs 100) against all protein sequences in the complete qPTMplants database. All pairwise sequence alignment results were tested for each query to identify matches with experimentally derived phosphosites. All query phosphosites with matches to the qPTMplants database were labeled as inferred positives, and the matches were compiled as a list to improve phosphosite annotation and provide additional supportive information.

### 5.5 Data and code availability

A detailed markdown page provides explanations for all analysis steps, code for reproducing results and figures, and links to raw data and results, which are available on GitHub at https://github.com/eporetsky/PhosBoost. The protein phosphorylation data used in this study is available for download directly from the qPTMplants database (Xue et al., 2022) and PhosphoLingo GitHub repository (Zuallaert et al., 2022). Additionally, we assembled several helper functions to facilitate raw data processing and conversion between different file formats used by different protein phosphorylation prediction methods as a python package named PTMtools that is available through the official PyPI repository.

## AUTHOR CONTRIBUTIONS

EP, CMA, and TZS designed the research. EP performed the research and drafted the manuscript. EP, CMA, and TZS revised and finalized the manuscript.

## CONFLICT OF INTEREST STATEMENT

The Authors did not report any conflict of interest.

## Supporting information


**Figure S1.** **Overview of the complete protein phosphorylation data available at the qPTMplants database and PhosBoost performance. (A)** A bar graph showing the aggregated number of unique phosphosites for each species identified in all included experiments on a log10 scale. Bars were colored by the plant family to which the species belongs. **(B)** An aggregation of all unique phosphoproteins in select species combined by individual datasets available for each species, sorted from largest to smallest datasets. **(C)** An aggregation of all unique phosphosites in select species combined by individual datasets available for each species, sorted from largest to smallest datasets.

**Figure S2.** **Overview of the label imbalance in the *A*** ***. thaliana*** **qPTMplants and Ramasamy22 datasets.** The total number of **(A)** negative and **(B)** positive phosphosites in the combined *A. thaliana* qPTMplants and Ramasamy22 datasets for each of the Ser, Thr, and Tyr residues were plotted as a bar graph. **(C)** The ratios between the number of positive phosphosites and the total number of phosphosites in the *A. thaliana* qPTMplants and Ramasamy22 datasets were calculated for each of the Ser, The, and Tyr residues and plotted as a bar graph.

**Figure S3.** **Comparison of the PhosBoost stacking classifier performance with the individual CatBoost classifiers using the Ramasamy22 dataset. (A‐C)** Comparison of the performance of the two independent CatBoost (CB) classifiers trained with equal class weights (orange), balanced class weights (green), and the PhosBoost stacking classifier (blue), showing the receiver operating characteristic curve and area under receiver operating characteristic curve (AUROC) score, precision‐recall curve and area under precision‐recall curves (AUPRC) score, true positive rate (TPR), false positive rate (FPR), and probability (P) threshold, for the Ser/Thr model, and similarly **(D‐F)** for the Tyr model.

**Figure S4.** **Comparing the predictive performance of PhosBoost with existing protein phosphorylation classification methods on the Ramasamy22 dataset. (A‐C)** Comparison of the performance results for DeepPhos (green), PhosphoLingo (blue), and PhosBoost (orange), showing the receiver operating characteristic curve and area under receiver operating characteristic curve (AUROC) score, precision‐recall curve and area under precision‐recall curves (AUPRC) score, true positive rate (TPR), false positive rate (FPR), and probability (P) threshold, and similarly **(D‐F)** for the Tyr model.

**Figure S5.** **Results of the PhosBoost Y model produce more informative predicted probability scores and achieve better performance when recall is prioritized. (A)** Confusion matrix for the Y model classification results for PhosBoost (N stands for non‐phosphorylated). **(B)** A split violin plot showing the distribution of the predicted probability values for all true positive (TP) and false positive (FP) samples (predicted probability > .5) for the tyrosine (Y) phosphosites predicted by PhosBoost. **(C)** Evaluation of the PhosBoost model performance using the Fβ measure at different β values. All results are based on the PhosBoost model trained on the *A. thaliana* qPTMplants dataset.

**Figure S6.** **Despite lower precision, the PhosBoost S/T and Y models trained on the Ramsamy22 dataset produce more informative predicted probability scores and achieve better performance when recall is prioritized. (A‐C)** Confusion matrices for the S/T model classification results and **(D‐F)** Y model classification results for PhosBoost, PhosphoLingo, and DeepPhos, respectively (N stands for non‐phosphorylated). **(G)** A split violin plot showing the distribution of the predicted probability values for all true positive (TP) and false positive (FP) samples (predicted probability > .5) separated by serines (S), threonines (T), and available tyrosines (Y) for PhosBoost, PhosphoLingo, and DeepPhos. **(H‐I)** Evaluation of the PhosBoost, PhosphoLingo, and DeepPhos model performances using the Fβ measure at different β values, in the S/T and Y models, respectively. All results are based on models trained on the Ramasamy22 dataset.

**Figure S7.** **Using a DIAMOND‐based pairwise alignment analysis improves Tyr phosphosite annotation and reduces false positive label uncertainty. (A)** A confusion matrix using a DIAMOND‐based binary protein phosphorylation prediction was trained and tested on the *A. thaliana* qPTMplants Tyr phosphosite data. **(B)** Confusion matrix for the PhosBoost Y model results after combining true positive and inferred positive (IP) phosphosites accounting for the false positive (FP) phosphosites. **(C)** A split violin plot showing the distribution of the predicted probability values for all FP and IP Tyr phosphosites (predicted probability > .5) for the PhosBoost results. In all confusion matrices, N stands for non‐phosphorylated.

**Table S1.** Hyper‐parameters obtained following 100 iterations using the Bayesian optimization bayes_opt Python package while optimizing for F1 score.
**Table S2.** Summary of the gene IDs and site coordinates of a small number of verified functional *A. thaliana* phosphosites in the brassinosteroid, ethylene, and abscisic acid phytohormone pathways and the matching phosphosites in the top blast hit in *Zea mays* and *Triticum aestivum* with the predicted probability scores for PhosBoost and PhosphoLingo. The top predicted probability score is marked in bold and non‐conserved sites were left blank.
