# Supplementary figures and images for: PhosBoost: Improved phosphorylation prediction recall using gradient boosting and protein language models
Source: Plant Direct. 2023 Dec 20;7(12):e554. doi: 10.1002/pld3.554 (PMC10732782; doi:10.1002/pld3.554)

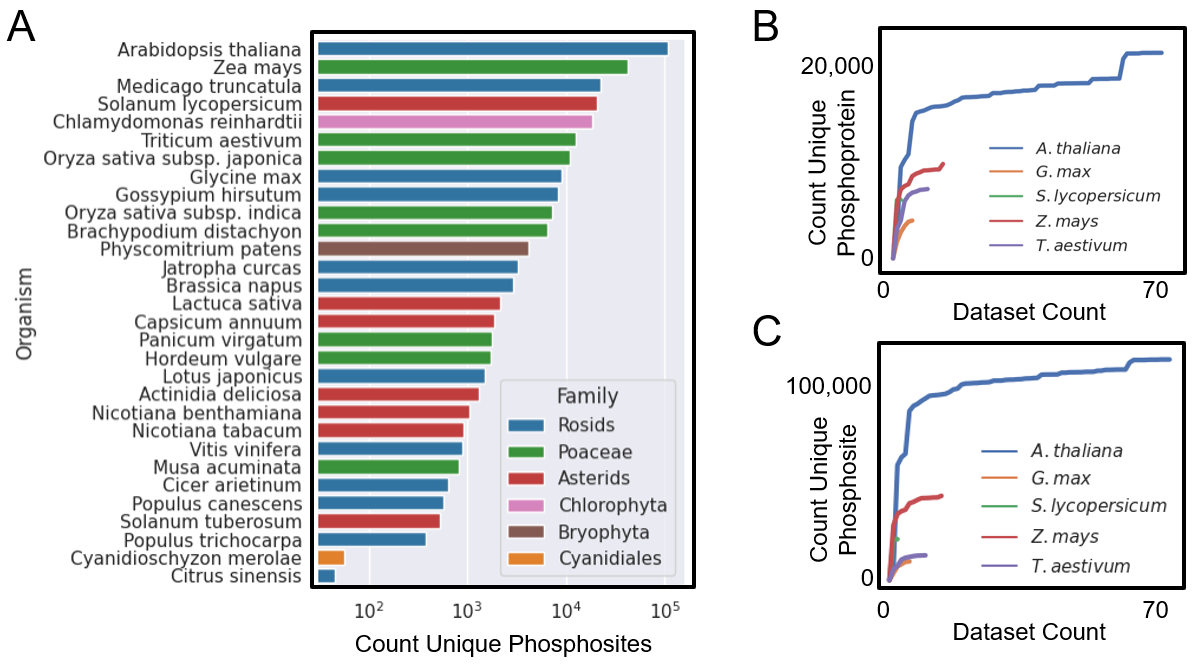

Supplement: Supplementary file 1 — Figure S1. Overview of the complete protein phosphorylation data available at the qPTMplants database and PhosBoost performance. (A) A bar graph showing the aggregated number of unique phosphosites for each species identified in all included experiments on a log10 scale. Bars were colored by the plant family to which the species belongs. (B) An aggregation of all unique phosphoproteins in select species combined by individual datasets available for each species, sorted from largest to smallest datasets. (C) An aggregation of all unique phosphosites in select species combined by individual datasets available for each species, sorted from largest to smallest datasets. [file PLD3-7-e554-s006.png]

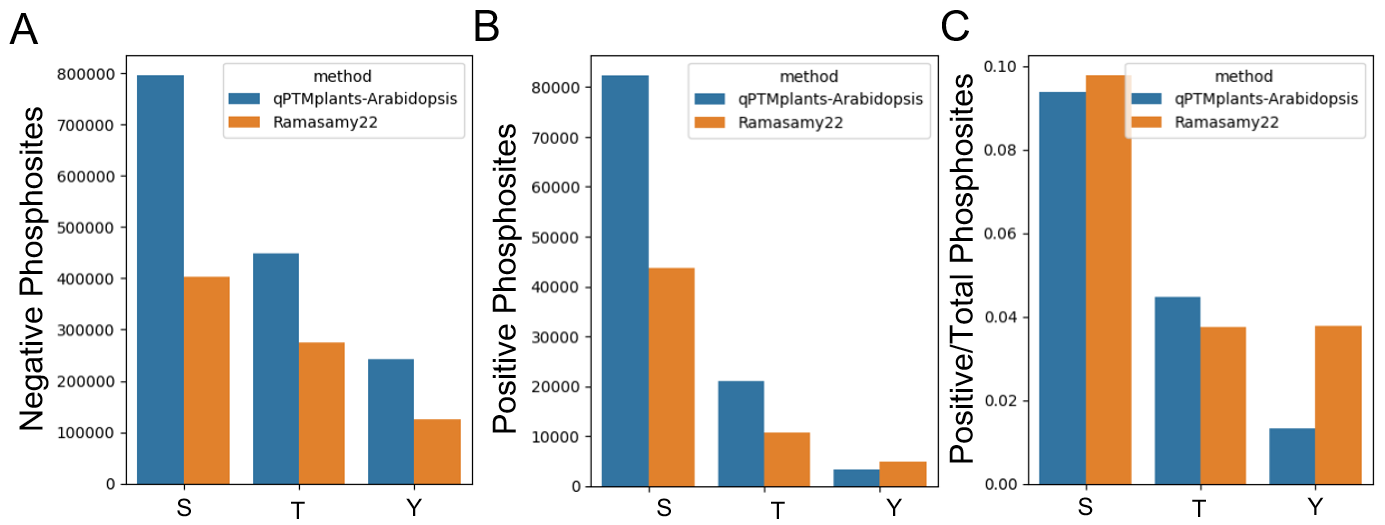

Supplement: Supplementary file 2 — Figure S2. Overview of the label imbalance in the A . thaliana qPTMplants and Ramasamy22 datasets. The total number of (A) negative and (B) positive phosphosites in the combined A. thaliana qPTMplants and Ramasamy22 datasets for each of the Ser, Thr, and Tyr residues were plotted as a bar graph. (C) The ratios between the number of positive phosphosites and the total number of phosphosites in the A. thaliana qPTMplants and Ramasamy22 datasets were calculated for each of the Ser, The, and Tyr residues and plotted as a bar graph. [file PLD3-7-e554-s004.png]

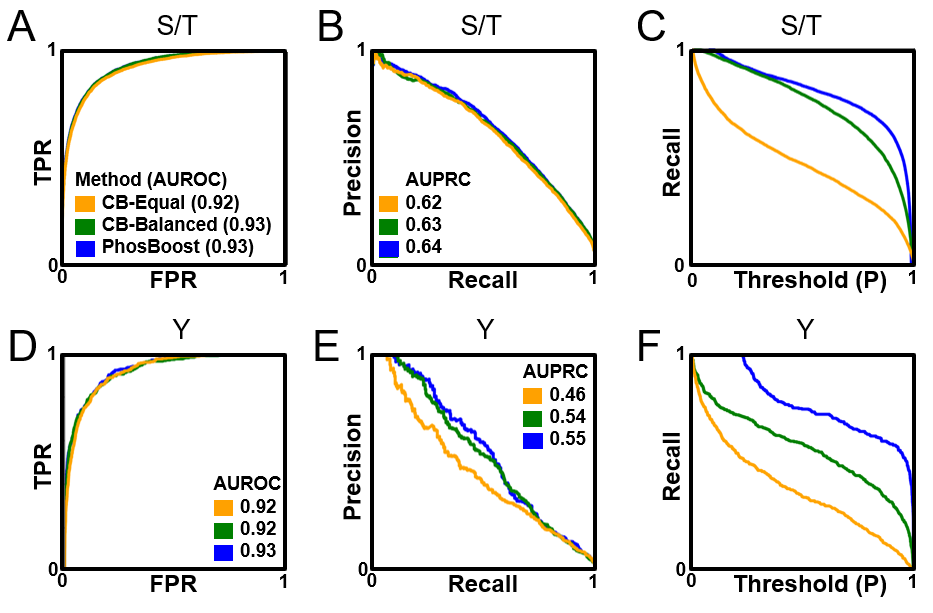

Supplement: Supplementary file 3 — Figure S3. Comparison of the PhosBoost stacking classifier performance with the individual CatBoost classifiers using the Ramasamy22 dataset. (A‐C) Comparison of the performance of the two independent CatBoost (CB) classifiers trained with equal class weights (orange), balanced class weights (green), and the PhosBoost stacking classifier (blue), showing the receiver operating characteristic curve and area under receiver operating characteristic curve (AUROC) score, precision‐recall curve and area under precision‐recall curves (AUPRC) score, true positive rate (TPR), false positive rate (FPR), and probability (P) threshold, for the Ser/Thr model, and similarly (D‐F) for the Tyr model. [file PLD3-7-e554-s005.png]

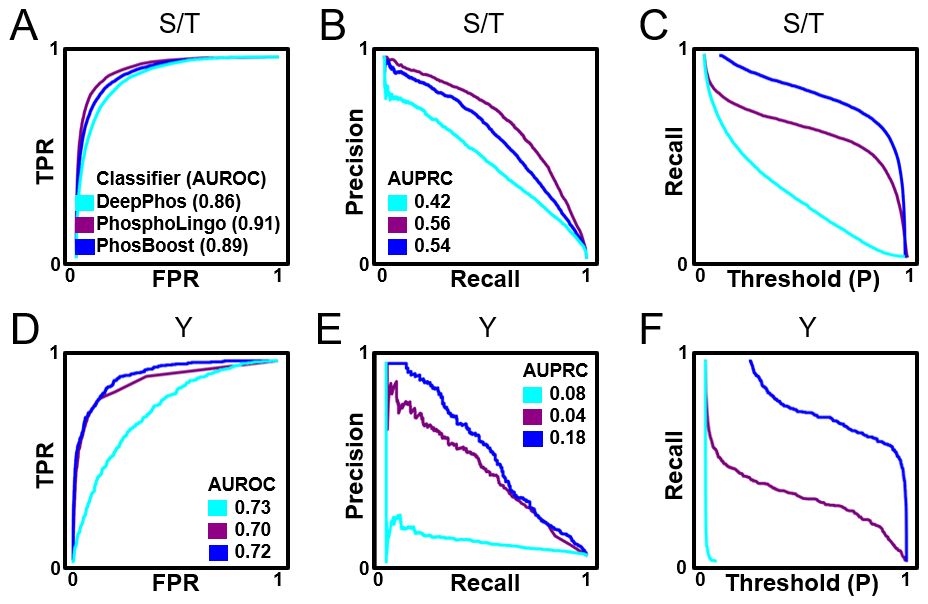

Supplement: Supplementary file 4 — Figure S4. Comparing the predictive performance of PhosBoost with existing protein phosphorylation classification methods on the Ramasamy22 dataset. (A‐C) Comparison of the performance results for DeepPhos (green), PhosphoLingo (blue), and PhosBoost (orange), showing the receiver operating characteristic curve and area under receiver operating characteristic curve (AUROC) score, precision‐recall curve and area under precision‐recall curves (AUPRC) score, true positive rate (TPR), false positive rate (FPR), and probability (P) threshold, and similarly (D‐F) for the Tyr model. [file PLD3-7-e554-s001.png]

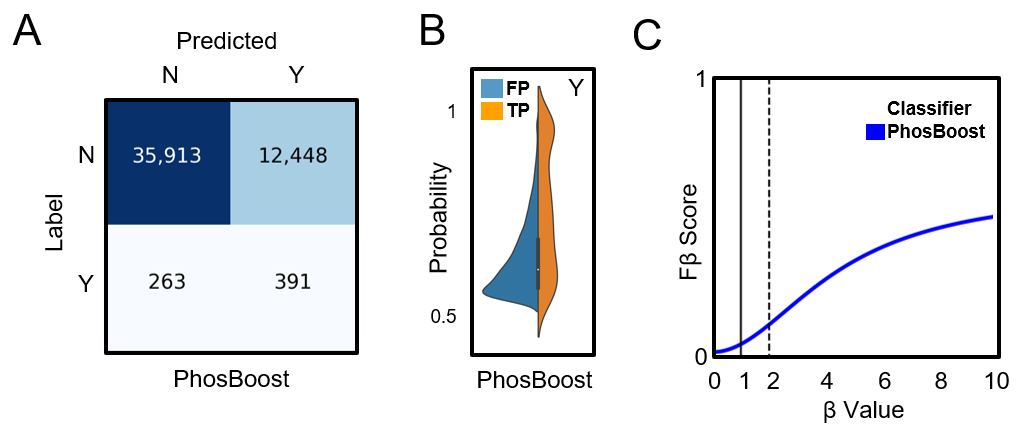

Supplement: Supplementary file 5 — Figure S5. Results of the PhosBoost Y model produce more informative predicted probability scores and achieve better performance when recall is prioritized. (A) Confusion matrix for the Y model classification results for PhosBoost (N stands for non‐phosphorylated). (B) A split violin plot showing the distribution of the predicted probability values for all true positive (TP) and false positive (FP) samples (predicted probability > .5) for the tyrosine (Y) phosphosites predicted by PhosBoost. (C) Evaluation of the PhosBoost model performance using the Fβ measure at different β values. All results are based on the PhosBoost model trained on the A. thaliana qPTMplants dataset. [file PLD3-7-e554-s007.png]

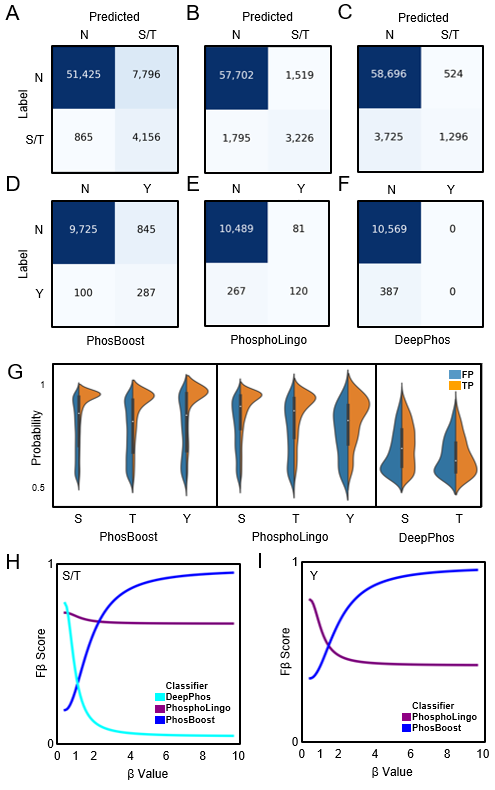

Supplement: Supplementary file 6 — Figure S6. Despite lower precision, the PhosBoost S/T and Y models trained on the Ramsamy22 dataset produce more informative predicted probability scores and achieve better performance when recall is prioritized. (A‐C) Confusion matrices for the S/T model classification results and (D‐F) Y model classification results for PhosBoost, PhosphoLingo, and DeepPhos, respectively (N stands for non‐phosphorylated). (G) A split violin plot showing the distribution of the predicted probability values for all true positive (TP) and false positive (FP) samples (predicted probability > .5) separated by serines (S), threonines (T), and available tyrosines (Y) for PhosBoost, PhosphoLingo, and DeepPhos. (H‐I) Evaluation of the PhosBoost, PhosphoLingo, and DeepPhos model performances using the Fβ measure at different β values, in the S/T and Y models, respectively. All results are based on models trained on the Ramasamy22 dataset. [file PLD3-7-e554-s002.png]

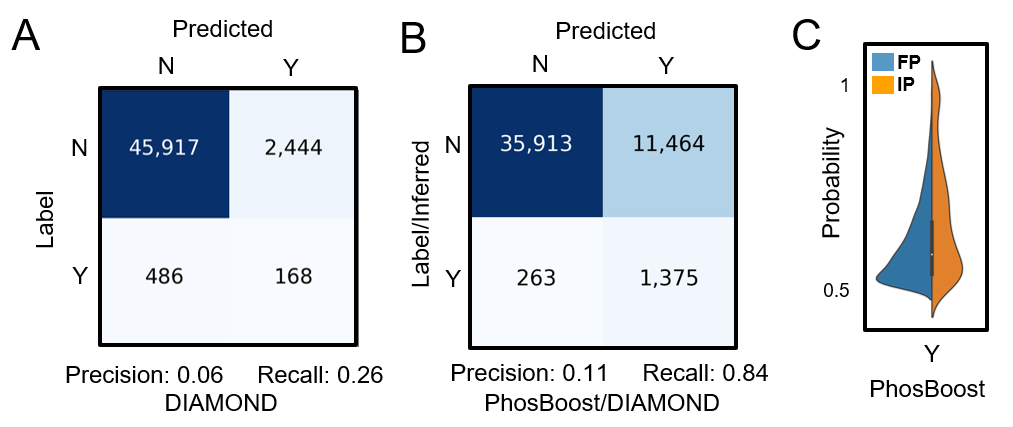

Supplement: Supplementary file 7 — Figure S7. Using a DIAMOND‐based pairwise alignment analysis improves Tyr phosphosite annotation and reduces false positive label uncertainty. (A) A confusion matrix using a DIAMOND‐based binary protein phosphorylation prediction was trained and tested on the A. thaliana qPTMplants Tyr phosphosite data. (B) Confusion matrix for the PhosBoost Y model results after combining true positive and inferred positive (IP) phosphosites accounting for the false positive (FP) phosphosites. (C) A split violin plot showing the distribution of the predicted probability values for all FP and IP Tyr phosphosites (predicted probability > .5) for the PhosBoost results. In all confusion matrices, N stands for non‐phosphorylated. [file PLD3-7-e554-s003.png]
